# Acute changes of pro-inflammatory markers and corticosterone in experimental subarachnoid haemorrhage: A prerequisite for severity assessment

**DOI:** 10.1371/journal.pone.0220467

**Published:** 2019-07-30

**Authors:** Annika Bach, Catharina Conzen, Gerrit Alexander Schubert, Christian Bleilevens, Ute Lindauer

**Affiliations:** 1 Translational Neurosurgery and Neurobiology, University Hospital Aachen, RWTH Aachen, Aachen, Germany; 2 Department of Neurosurgery, University Hospital Aachen, RWTH Aachen, Aachen, Germany; 3 Department of Anaesthesiology, University Hospital Aachen, RWTH Aachen, Aachen, Germany; University of PECS Medical School, HUNGARY

## Abstract

Many details of the pathophysiology of subarachnoid haemorrhage (SAH) still remain unknown, making animal experiments an indispensable tool for assessment of diagnostics and therapy. For animal protection and project authorization, one needs objective measures to evaluate the severity and burden in each model. Corticosterone is described as a sensitive stress parameter reflecting the acute burden, and inflammatory markers can be used for assessment of the extent of the brain lesion. However, the brain lesion itself may activate the hypothalamic-pituitary-adrenal-axis early after SAH, as shown for ischemic stroke, probably interfering with early inflammatory processes, thus complicating the assessment of severity and burden on the basis of corticosterone and inflammation. To assess the suitability of these markers in SAH, we evaluated the courses of corticosterone, IL-6 and TNF-α up to 6h in an acute model simulating SAH in continuously anaesthetized rats, lacking the pain and stress induced impact on these parameters. Animals were randomly allocated to sham or SAH. SAH was induced by cisterna magna blood-injection, and intracranial pressure and cerebral blood flow were measured under continuous isoflurane/fentanyl anaesthesia. Withdrawn at predetermined time points, blood was analysed by commercial ELISA kits. After 6h the brain was removed for western blot analysis of IL-6 and TNF-α. Serum corticosterone levels were low with no significant difference between sham and SAH. No activation of the HPA-axis was detectable, rendering corticosterone a potentially useful parameter for stress assessment in future chronic studies. Blood IL-6 and TNF-α increased in both groups over time, with IL-6 increasing significantly more in SAH compared to sham towards the end of the observation period. In the basal cortex, IL-6 and TNF-α increased only in SAH. The pro-inflammatory response seems to start locally in the brain, reflected by an increase in peripheral blood. An additional surgery-induced systemic inflammatory response should be considered.

## Introduction

Stroke is still the second most common cause of death worldwide and the third most common cause of disability [1]. Though the prevalence of ischemic stroke far exceeds that of haemorrhagic strokes, the subgroup of patients with subarachnoid haemorrhage in particular gains attention due to its significantly higher number of deaths in comparison to ischemic stroke [1]. Additionally, patients suffering from SAH are younger than those suffering from ischemic stroke [2].

Recently, early brain injury in particular has been identified as a major contributor to overall outcome after SAH. Despite considerable research efforts, however, the underlying pathophysiology is largely unknown, but may hold the key to a more tailored and hopefully more effective treatment approach. Within the last years, it has become clear that the very early events starting from bleeding onset are responsible for the further development of the disease and thus also provide the basis for the detrimental cascade of delayed events like delayed cerebral ischemia (DCI) and tissue infarction [3]. One important element of early brain injury (EBI) is the inflammatory response within the tissue that is followed by systemic inflammation in most patients suffering from SAH (see reviews [3–5]). During this inflammatory response, increasing concentrations of pro-inflammatory cytokines can be found in patients- and rat-blood plasma-, and cerebrospinal fluid-samples [6–9]. Especially interleukin 6 (IL-6) and tumour necrosis factor alpha (TNF-α) are discussed to contribute to detrimental vascular events in the delayed phase of SAH [7, 8, 10]. Molecules produced and released during inflammatory processes may therefore serve as reliable biomarkers for the assessment of the extent of the brain lesion.

In addition to inflammatory processes, neuroendocrine dysfunction has been described as a stress response to brain injury. In preclinical research studies modelling global or focal ischemia it has been shown that the lesion itself may lead to an activation of the hypothalamic-pituitary-adrenal (HPA)-axis [11–13], adding to the psychogenic stress response under conscious experience of the acute events of the disease. In addition, a significantly elevated level of cortisol at the day of SAH at admission has been described in patients suffering from SAH [14]. It is well known that an HPA-axis activation increases blood glucocorticoid levels resulting in suppression of the systemic immune system. It is therefore important to consider a possible influence of common glucocorticoid stress regulators (for example corticosterone) on the immune system before suggesting cytokines as useful biomarkers for brain lesion assessment.

Besides being an indicator of brain lesion induced immunosuppression, corticosterone is also a sensitive parameter for psychogenic stress pointing towards harm and burden, an aspect especially important with regard to animal experimentation [15]. As the patients’ admission to the hospital and consequently the inclusion to studies occur delayed in respect to the initial event, animal experiments modelling SAH are still needed to gain basic knowledge of the early injury within the first few hours after the onset of bleeding. With regard to animal protection, severity assessment is a fundamental prerequisite for project authorization (see EU directive 2010/ 63/ EU). In this context, the well-established and sensitive stress parameter corticosterone often serves as a reliable measure of severity [15]. However—as pointed out above—in the field of stroke research, corticosterone may not be used unconditionally as a marker of burden and suffering of the animal due to the possibility of its brain lesion induced activation and release and thus not necessarily indicating suffering of the animal.

In this study, by applying the widely used SAH blood-injection model in rats [16–18], we therefore tested the hypothesis of an early HPA axis activation in SAH which may–in addition–have suppressive effects on the pro-inflammatory cytokines IL-6 and TNF-α in blood compared to brain tissue, as already shown for ischemic stroke at later time points. Therefore, we further aimed to show whether it is yet possible to detect a brain-lesion induced increase of pro-inflammatory markers in the systemic circulation, thus making them usable screening parameters in blood samples reflecting tissue damage. To our knowledge this is the first study to show time courses of two main pro-inflammatory markers combined with analyses of these markers in the brain tissue, complemented with analysis of blood corticosterone in the same experimental setup.

Blood samples were analysed up to 6 hours (h) after SAH in continuously anesthetized rats, a condition where the pain and conscious psychogenic stress induced impact on these parameters is reliably eliminated.

The findings of this study will provide a well-grounded basis for application of these measures for brain lesion estimation as well as for severity assessment during chronic progression in the awake animal following SAH induction.

## Methods

### Animals

All experiments were conducted in accordance with the German Federal Law regarding the protection of animals and the DIRECTIVE 2010/63/EU on the protection of animals used for scientific purpose. The governmental care and use committee (LANUV, Recklinghausen, NRW, Germany) granted official permission (file reference: 84–02.04.2015.A412).

A total of 32 male Wistar rats (Janvier Labs, France) weighing between 320 +/- 20g (range 287-357g) were used, divided in SAH or sham with n = 16 in each group. The animals were kept in the central facility within the Institute for Laboratory Animal Science (University Hospital Aachen; certified according to DIN ISO 9001/2015 QM) and group-housed in Type 2000 rat filter top cages (Tecniplast, Hohenpeißenberg, Germany) under specific pathogen free conditions according to the guidelines of the Federation of European Laboratory Animal Science Associations [19]. The cages contained bedding material (Holzgranulat ¾ S, Rettenmeier, Germany) and nestlets (Plexx; Elst, Netherlands). Room temperature (22±2°C) and humidity (55±5%) were controlled, the light-dark cycle was set at 12h (lights on from 7:00 a.m. to 7:00 p.m.). Food (V1534-300, Ssniff, Soest, Germany) and water were given ad libitum. After supply from the breeder, animals were allowed to habituate to the new surrounding for at least 7 days.

No data on the variance of the parameters to be measured existed beforehand, therefore a marker-specific a-priori sample size calculation was not possible. We therefore performed our study with numbers usually included in this kind of analysis [9, 20].

### Surgery and recording phase

**Fig 1:**
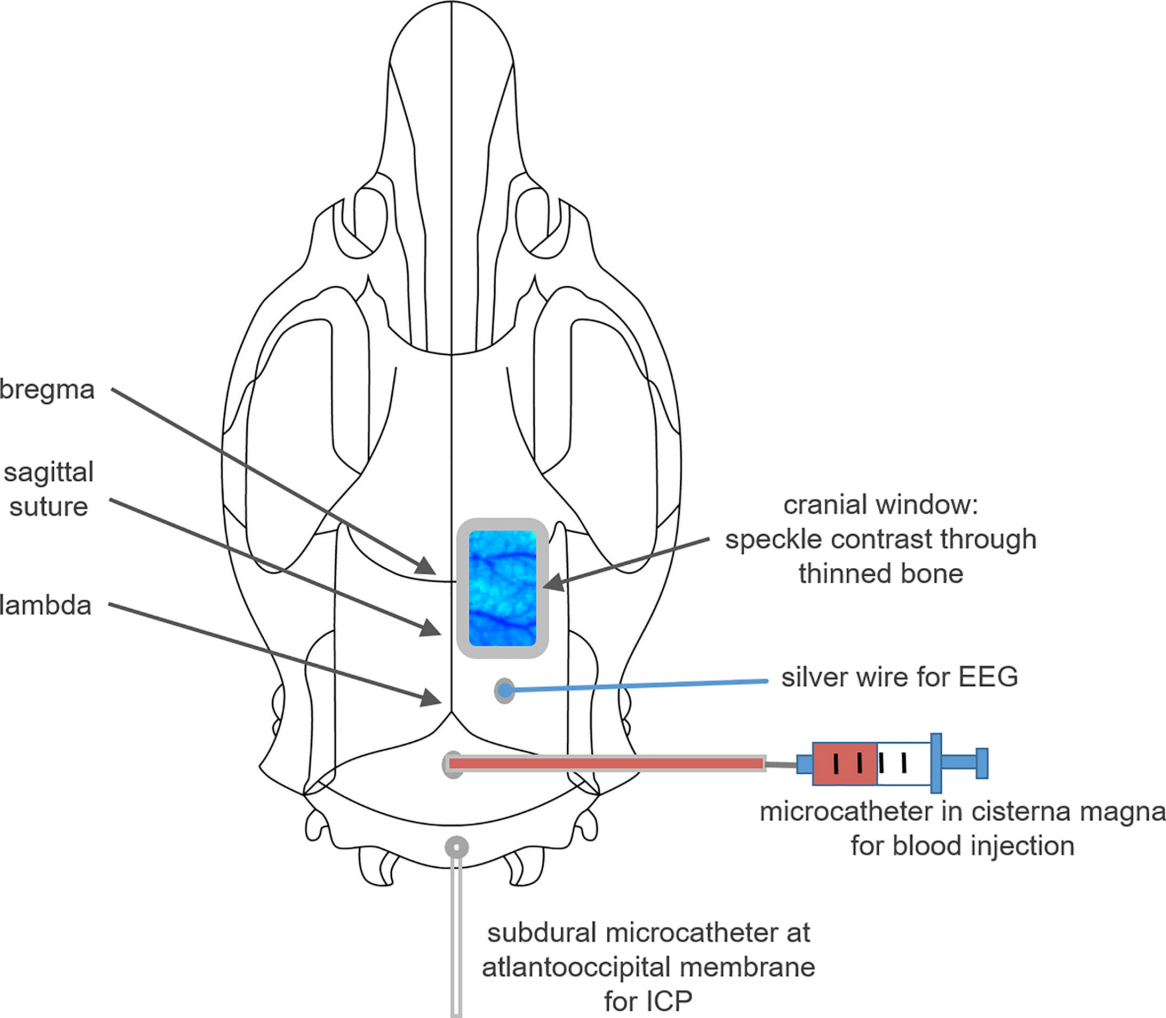
Illustration of the experimental setup. The positions of the cranial window for laser speckle contrast imaging, the silver wire for EEG recording and the catheters for blood injection and ICP assessment are shown.

Surgery and recording setup was conducted as previously described [21]. Briefly, anaesthesia was induced in the morning (7:45–8:00 am) using Isoflurane (Isoflurane, Forene, Ludwigshafen, Germany), intravenous fentanyl infusion and local anaesthesia at each skin incision (Ropivacainhydrochlorid, 2mg/ml, Fresenius Kabi, Bad Homburg, Germany) were used as analgesia. The animal was tracheotomised and mechanically ventilated. Body temperature was monitored and regulated by a serve-controlled heating pad (Harvard Apparatus Ltd., Kent, England). Arterial blood pressure, heart rate, oxygen saturation and blood gases were monitored, too. A small silver wire for cortical electroencephalic recording was positioned onto the dura with a reference electrode placed onto the neck musculature. A bone window was drilled over the right parietal cortex for measurement of cerebral blood flow (CBF), and ICP was measured using a fluid-filled microcatheter, inserted into the atlantookzipital membrane. SAH was induced via injection of 500μl blood within one minute, using a second microcatheter inserted into the cisterna magna. In contrast to the common approach in this model, we abstained from heightening the lower body of the animals to prevent moving artefacts of CBF recordings. Therefore a slightly higher failure rate of SAH induction occurred due to blood distribution towards the spinal cord, resulting in more animals to be excluded while presenting smaller or even absent blood coverage at the basal brain surface. Sham animals underwent the same procedure as SAH animals except the blood injection (Fig 1).

CBF (by laser speckle contrast analysis) and haemoglobin oxygenation (by optical spectroscopy) were recorded under resting conditions (baseline and sham-group) and for 6h after blood-injection with animals positioned under a prototype superficial tissue imaging system (STIS, Biomedical Optics Laboratory, Rheinahrcampus Remagen, Germany). Additionally, systemic arterial blood pressure (ABP), intracranial pressure (ICP) and EEG were recorded continuously. Blood gases were evaluated regularly, and adequate depth of anaesthesia was confirmed by regularly checking for loss of flexor and toe pinch reflexes.

Six hours after blood injection, the animal was sacrificed in deep anaesthesia by an intravenous (i.v.) injection of highly concentrated potassium chloride (2.5M) (~ 05:00 p.m., range: 04:45 p.m.– 05:54 p.m.) The brain was removed, rinsed with saline for removal of epidurally adherent blood, and SAH was acutely graded according to our modified SAH scale [21]. Pictures were taken for blinded offline confirmation of the acute grading (S1 Fig). After scoring, the hemispheres were separated, with the right hemisphere transferred in -20°C methylbutane for 10min prior to storage at -80°C and the left hemisphere directly frozen and stored at -80°C for further analysis via western blot.

Processing of the recorded data was performed as already described elsewhere [21].

### Experimental design

**Fig 2:**
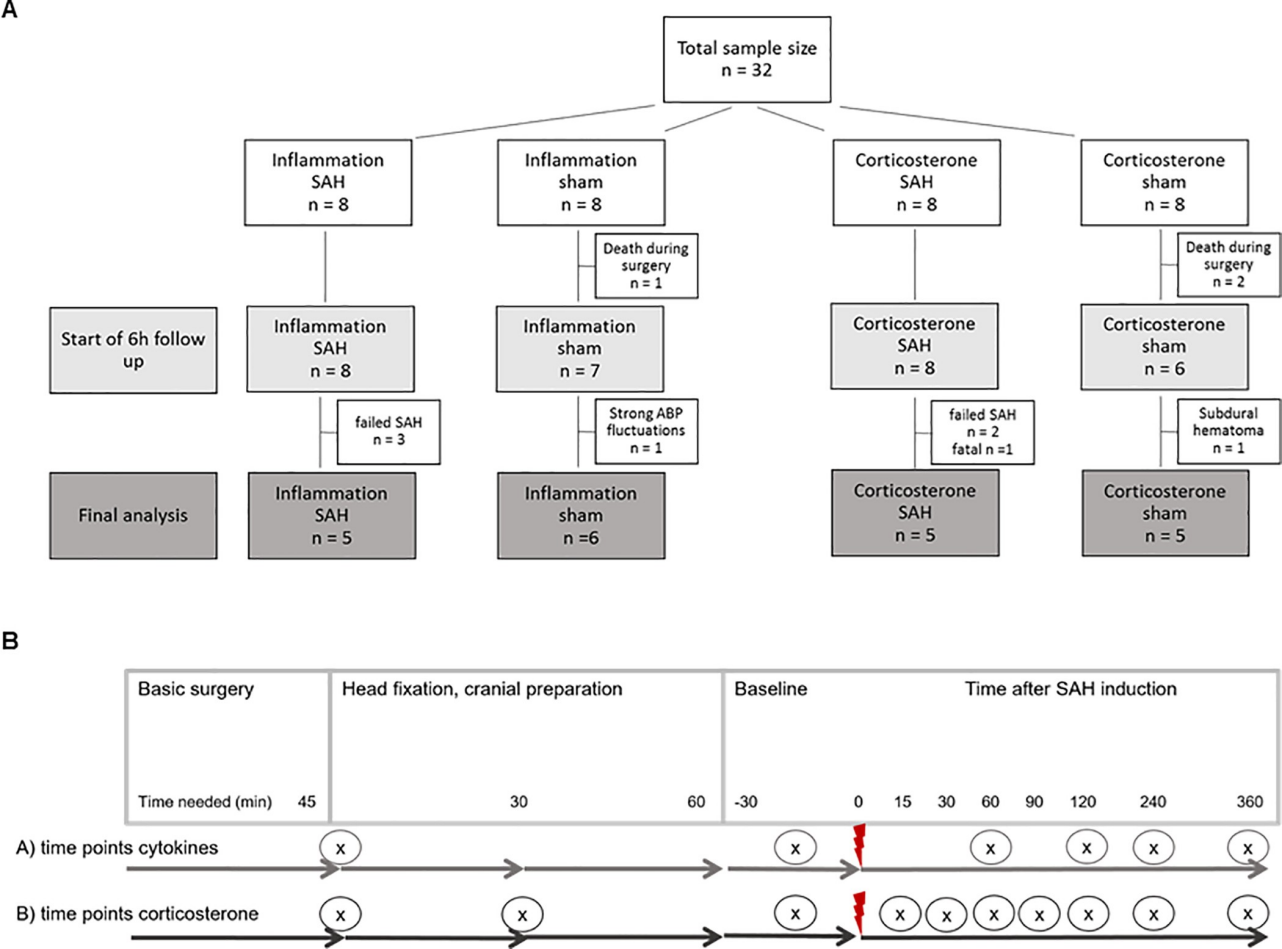
Experimental design. A: Diagram of sample size distribution: 32 animals were randomly allocated to four different groups for blood sampling for inflammation markers (IL-6 and TNF-α) and corticosterone; after subtracting drop-outs from surgery and recording phase, a total of 21 animals were included in the final analysis. Subarachnoid hemorrhage (SAH) was induced by injection of 500μl arterial blood within 1 minute into the cisterna magna, sham animals did not receive any injection. B: Time points for blood sampling for inflammatory cytokine and corticosterone analysis: The diagram shows the time points (x) when blood was withdrawn within basic surgery (anaesthesia, tracheotomy, ventilation, A./V. femoralis cannulation), head fixation and cranial preparation (turning to prone position, fixation with ear bars in stereotactic frame, cranial window preparation, trepanations for EEG and ICP recording, cannula for icv blood injection) followed by the measurement period; red flash marks the time point of SAH induction.

The measurement of pro-inflammatory cytokines as well as corticosterone in the same animal for all predetermined time points was not possible due to the limited amount of blood available for in-vivo sampling. Two groups with 16 animals per group for pro-inflammatory cytokine or corticosterone assessment, respectively, were scheduled. The total of 32 animals were randomly allocated to the four different groups for blood sampling for inflammation markers (IL-6 and TNF-α) and corticosterone; after subtracting drop-outs from surgery and recording phase, a total of 21 animals were included in the final analysis (Fig 2A). Subarachnoid hemorrhage (SAH) was induced by injection of 500μl arterial blood within 1 minute into the cisterna magna, sham animals did not receive any injection.

Starting within the surgery period and continued during the 6h recording period, blood samples were withdrawn at 9 time points for corticosterone analysis and at 5 time points for cytokine assessment (Fig 2B), with approximately 80μl (corticosterone) or 200μl (cytokines) collected in a serum tube at each time point. At the end of the recording period, (time point 10 in corticosterone groups, time point 6 in inflammatory cytokine groups) a final exsanguination with withdrawal of 6-10ml arterial blood was performed. For corticosterone analysis, blood was kept at room temperature for 60min until clotting, followed by centrifugation at 3500rpm for 10minutes at 4°C (Eppendorf 5424R, Eppendorf AG, Hamburg, Germany). For cytokine analysis, blood samples were immediately centrifuged at 1000xg for 20minutes at room temperature (Eppendorf 5424R, Eppendorf AG, Hamburg, Germany). Serum or plasma was then stored at -80°C for later analysis.

### Blood analysis by ELISA

For the analysis of serum corticosterone, a commercial corticosterone rat/mouse ELISA kit was used (DEV9922, demeditec diagnostics, Kiel, Germany) according to the manufacturer’s manual. The final absorbance of the colorimetric reaction was determined by a microplate reader (Synergy HT Multi-Mode Microplate Reader; BioTek, Winooski, USA) at 450nm, and concentrations were calculated after blank correction using the simultaneously assessed concentration curve from standard samples.

To analyse IL-6 and TNF-α in the plasma, commercial quantikine ELISA kits were used (R6000B for IL-6, RTA00 for TNF-α, R&D, Minneapolis, USA), again according to the manufacturer’s manual. 100μl Standard, control and samples were incubated in duplicates for 2h with 50μl of assay diluent. Samples required a 2-fold dilution because of the low amount of sample (50μl sample + 50μl Calibrator Diluent). After adding 100μl of stop solution the optical density was determined by using a microplate reader (Synergy HT Multi-Mode Microplate Reader; BioTek, Winooski, USA) set to 450nm with wavelength correction set to 540nm, and the concentrations were calculated after blank correction.

### Western blot

For western blot analysis the left hemisphere of each brain was placed in a cooled brain matrix and cut in 7 slices of 2mm each. Tissue was collected from 3–4 sequential slices with regions of interest gained from Paxinos and Watson (Paxinos, George; Watson, Charles; 2007: The Rat Brain in Stereotaxic Coordinates. London: Academic Press, 6^th^ edition). For samples from the parietal cortex, slices 2–5 were used, tissue from the basal cortex was collected from slices 3–6 and for hippocampus slices 4–6 were used (S2 Fig). The amount of 34 ± 5 mg of each brain tissue sample were homogenized on ice for ~ 30 seconds in 800μl RIPA buffer before centrifugation (2 min at 4k rpm and 4°C) through a shredder column (QIA Shredder; Qiagen inc. Hilden; Germany). The clear homogenate was stored at -80°C until final analysis. 5μl of each lysate were separated by SDS-Page on 10% acrylamide gels (TGX stain free; BioRad inc.; Düsseldorf; Germany). The stain-free technology enables fluorescent visualization of the separated proteins after photoactivation directly in the gel, as the proteins bound automatically to fluorophores within the gel during the separation process. The visualized total protein amount was used for later normalization of the target proteins. Proteins were transferred from the SDS Gel onto a polyvinylidene fluoride (PVDF) membrane (BioRad inc.). Membranes were incubated overnight at 4°C with appropriate primary antibody (IL-6: #NB600-1131, Novus biologicals, Colorado, USA; TNF-α: #AB66579, Abcam, Cambrige, United Kingdom) and subsequently with the suitable, horseradish-peroxidase conjugated secondary antibody (anti-rabbit, #7074, cell-signalling technology, Danvers MA, USA). Proteins were visualized by enhanced chemiluminescence using peroxidase substrate (Clarity Western ECL Blotting Substrate) and analysed using the ChemiDoc System with the Image Lab Software (BioRad laboratories, Germany). The software enables densitometric analysis of the target protein bands, and the total protein amount which was visualized by stain-free technology. The density of the target protein bends was normalized to the total protein amount. To avoid any interference of a possible, haemorrhage induced regulation of the expression of a house-keeping protein, we decided to use normalization to total protein only.

### Statistical analysis

All analyses as well as figure design were performed using GraphPad Prism 7.04 (GraphPad Software, Inc., La Jolla, USA). Normal distribution was tested using Shapiro-Wilk normality tests. Differences between groups as well as within groups were analysed using a repeated measures 2-way ANOVA followed by Sidak´s (comparisons between the groups), or Tukey´s (comparisons within the group) multiple comparisons tests. For analyses of the ELISA data multiple comparisons within the group were done comparing each mean with every other mean. For better overview, in the figures only significances in respect to baseline are depicted. For western blot analyses 2 way ANOVA was used, followed by Tukey´s (within group) or Sidak´s (between groups) multiple comparisons tests. In the figures, quantitative data are shown as box plots with median and 25% and 75% percentile as boxes and minimum to maximum values as whiskers, sometimes additionally all points are depicted. In the text, results are reported as median, [25%-75% percentile] and p-value. Statistical significance was set at p < 0.05.

## Results

A total of 32 rats were randomly assigned to the different experimental groups. Criteria for a successful induction of SAH were defined as follows: decrease of CBF, increase of ICP, confirmation via blinded offline grading of the brains (S1 Fig). All 3 criteria needed to be fulfilled. 11 animals were excluded from analysis for different reasons: n = 5 due to unsuccessful SAH induction (no ICP increase, no change in CBF), n = 3 due to unexpected death during surgery, n = 1 due to strong unexplained ABP fluctuations, n = 1 due to a subdural hematoma resulting in an unwanted ICP increase in a sham animal, and one case of a fatal SAH. Final analysis, therefore, was performed on 21 animals (Fig 2A).

Arterial blood gases, pH and body temperature remained in physiological ranges during the whole surgery and measurement period (Table 1).

**Table 1:** Physiological parameters achieved from blood gas analysis, rectal temperature probe and pulse oximeter.

	Sham (n = 11)	SAH (n = 10)
**Measurement at baseline**		
pO_2_ (mmHg)	108 [99–122]	129 [109–140]
pCO_2_ (mmHg)	40.1 [36.5–42.5]	37.5 [36.8–41.4]
pH	7.42 [7.41–7.44]	7.42 [7.40–7.43]
ABP (mmHg)	78.0 [74.5–85.0]	75.0 [71.8–84.0]
BT (°C)	37.3 [37.1–37.7]	37.4 [37.3–37.6]
Bpm	362 [344–378]	375 [358–385]
SpO_2_	89 [87–93]	93 [89–96]
**Measurement at 6h (before euthanasia)**		
pO_2_ (mmHg)	119 [115–130]	114 [109–118]
pCO_2_ (mmHg)	36.3 [35.3–37.7]	36.4 [35.9–37.1]
pH	7.39 [7.38–7.40]	7.38 [7.37–7.39]
ABP (mmHg)	76.0 [68.0–80.5]	71.0 [63.0–90.0]
BT (°C)	37.3 [37.0–37.7]	37.7 [37.5–37.8]
bpm	379 [341–406]	413 [389–421]
SpO_2_	89 [87–93]	89 [88–90]

pO_2_: arterial partial pressure of oxygen; pCO_2_: arterial partial pressure of carbon dioxide; BT: body temperature; bpm: beats per minute = heart rate; SpO_2_: arterial oxygen saturation. Values are expressed as median and [1.quartile - 3.quartile].

### Intracranial pressure, cerebral blood flow, arterial blood pressure

Injection of blood led to a typical significant increase of ICP as well as a significant decrease of CBF in SAH animals compared to sham. In the animals of the SAH group, the blood injection-induced transient peak response of ICP elicited the well-known cushing reflex [21] (S3 Fig).

Already during the surgery, ABP was carefully checked for possible pain induced alterations induced by specific procedures within the surgery even if no active withdrawal response was noticeable. By this we identified a critical procedure during the fixation of the animal´s head in the stereotactic frame. The insertion of the ear bars led to a small but significant transient increase of blood pressure although the depth of anaesthesia was regarded as sufficient because no flexor reflex and toe pinch reflex were elicited (pre ear bar 82mmHg [74–86], post ear bar 96 [78–100], p = 0.022) (S4 Fig).

### Inflammatory markers TNF-α and IL-6 from ELISA of blood samples

**Fig 3:**
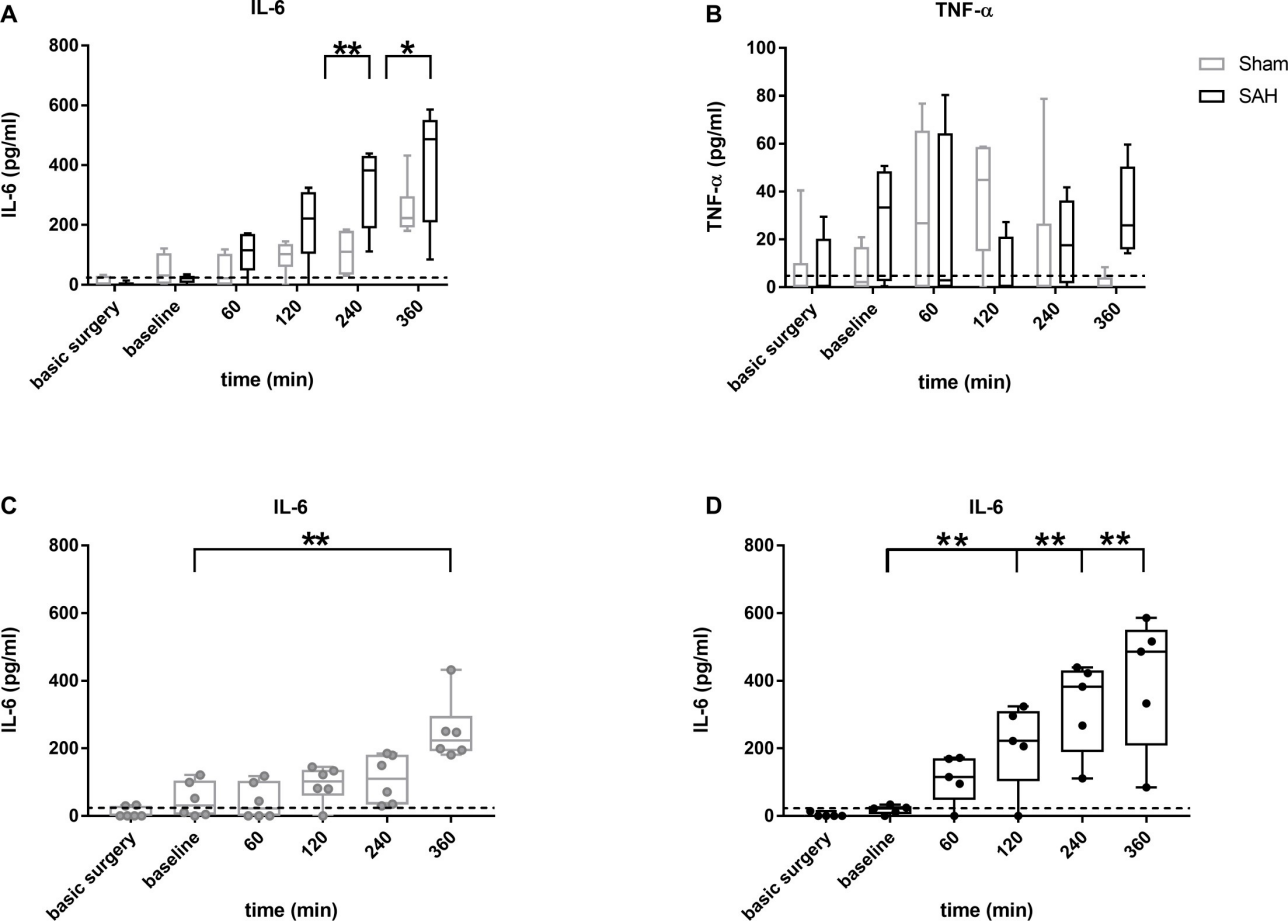
Box plots of IL-6 and TNF-α in blood samples from SAH (black) and sham (grey) animals. Data are presented as boxplot diagrams; the whiskers represent the minimum and maximum values, the dots show single points. Data were analyzed using repeated measures 2-way ANOVA followed by multiple comparisons tests: Sidak´s (comparisons between the groups; (A, B) IL-6 (A) and TNF-α (B) sham: n = 6; SAH: n = 5) or Tukey´s ((C, D) for IL-6, comparisons within the group; only significances in respect to baseline are depicted); * p < 0.05; ** p < 0.01; dotted line: minimum detectable dose (IL-6: 21pg/ml, TNF-α 5pg/ml).

For the two blood sampling time points during and after finishing the surgery (time points “basic surgery” and “baseline”), only very low concentrations were detected for the inflammatory markers IL-6 and TNF-α, respectively, representing a non-activated situation right before SAH induction (Fig 3).

Within the early recording phase at baseline and up to 2h later, TNF-α showed a very slight, albeit not significant, increase in sham as well as SAH animals, with normalization to baseline levels at the end of the observation period in sham animals (120min: 44.9 pg/ml [15.1–58.7]; 360min: 0.2 pg/ml [0–4.2], p = 0.020). In contrast to sham, SAH animals featured a secondary increase in TNF-α towards the end of the observation period (360min: 25.9 pg/ml [15.9–50.5], p = 0.174 SAH compared to sham). No significant difference for any other time point was detected when comparing sham with SAH (Fig 3B).

IL-6 showed stable resting (unstimulated) values with only a slight tendency towards higher values in sham animals up to 4h with a significant increase only at the end of the observation period (360min compared to each other time point: p<0.01; Fig 3C). After blood injection in SAH group animals, IL-6 remained at resting baseline values only up to 1h after injection followed by a continuous rise of IL-6 up to 4h after injection and stabilization at elevated levels for the remaining two hours (Fig 3D). At 4h and 6h after ictus, IL-6 was significantly higher in SAH animals (240min: sham 110.1 pg/ml [33.8–180.6]; SAH 382.4 pg/ml [189.2–430.8], p = 0.001; 360min: sham 223.2 pg/ml [191.4–295.9]; SAH 486.2 pg/ml [208.6–550.9], p = 0.045).

### Corticosterone from ELISA of blood samples

**Fig 4:**
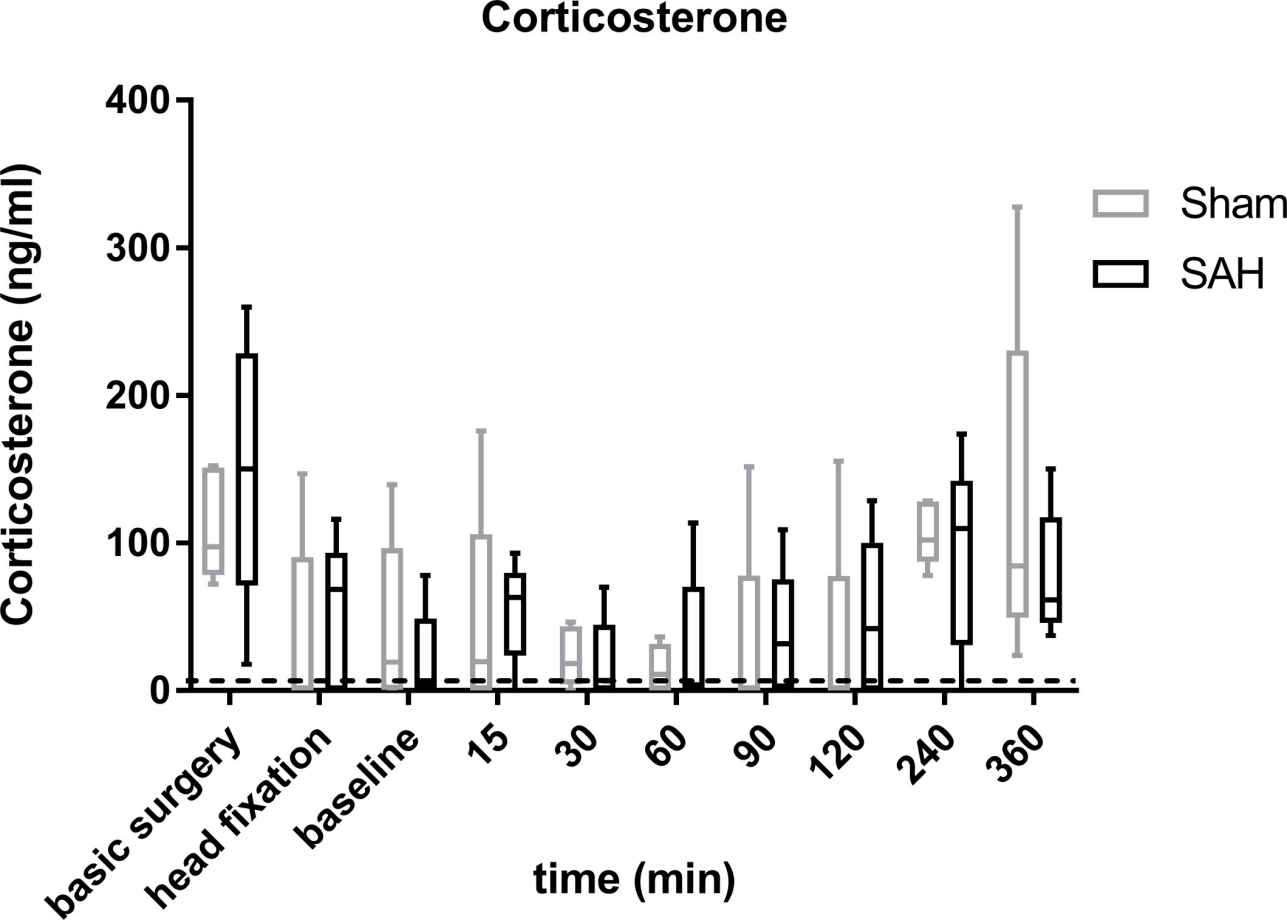
**Box plots of corticosterone in blood samples from SAH (black) and sham (grey)** Corticosterone was analysed at predefined sampling time points within the surgery and during the recording phase; Data are presented as boxplot diagrams; the whiskers represent the minimum and maximum values, the dots show single points. Data were analyzed using repeated measures 2-way ANOVA followed by Sidak´s multiple; SAH: n = 5, sham: n = 5; * p < 0.05; dotted line: minimum detectable dose 6.1ng/ml.

Corticosterone showed a u-shaped course in both groups with significantly higher concentrations at the first time point (= basic surgery) approximately 45 minutes after handling and anaesthesia induction (sham: 97.4 ng/ml [78.3–151.0]; 60min: 11.0 ng/ml [0–31.8]; p = 0.005; SAH: 150.2 ng/ml [71.3–228.7]; 60min: 3.9 ng/ml [0–70.3]; p<0.001), followed by reduced and very low levels at baseline and the first hours after injection with a moderate, secondary increase towards the end of the measuring period in the late afternoon / evening hours (60min versus 360min: sham: p<0.001; SAH p>0.05). Comparisons between sham and SAH revealed no significant difference at any time point (Fig 4). Additionally, even in one animal with a lethal course of SAH (not included in final analysis) we did not find exceeding values different to those from the other SAH animals (S5 Fig).

### Inflammatory markers TNF-α and IL-6 in brain tissue by Western blot analysis

**Fig 5:**
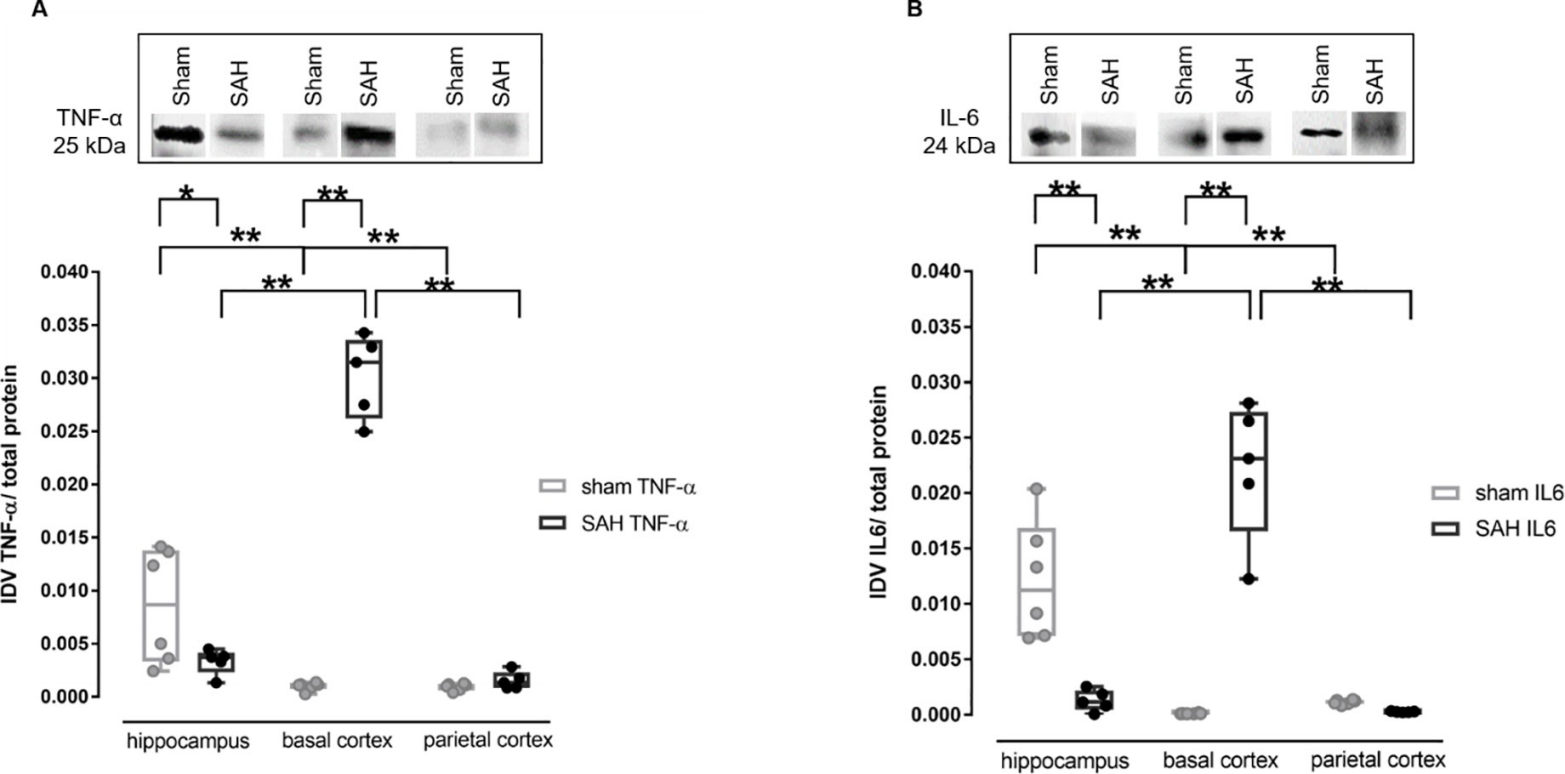
**Western blot analysis of TNF-α (A) and IL-6 (B) in 3 different brain regions** Upper part: representative protein bands detecting TNF-α or IL-6. Lower part: box plots of quantitative analysis from SAH (black) and sham (grey); data were analysed using 2 way ANOVA followed by Tukey´s (within groups) or Sidak´s (between groups) multiple comparisons tests. brain tissue was harvested after euthanasia at the end of the recording phase at 6h. * p < 0.05; ** p < 0.01.

Western blot analysis for TNF-α revealed a significant increase in the basal cortex of SAH animals in comparison to sham animals (SAH: 0.031 IDV [0.026–0.034], sham 0.0011 IDV [0.0007–0.0012], p < 0.001). Additionally, the sham animals showed higher levels of TNF-α in the hippocampus when compared to SAH animals (SAH: 0.00037 [0.0023–0.0045]; sham: 0.0087 [0.0033–0.0138], p < 0.01). In sham animals, the hippocampus showed significantly more TNF-α than the basal cortex and the parietal cortex (hippocampus: 0.0087 IDV [0.0033–0.0138]; basal cortex: 0.0011 IDV [0.0007–0.0012], p < 0.001; parietal cortex: 0.0011 IDV [0.0006–0.0012], p < 0.001), whereas in SAH animals, TNF-α was significantly highest in basal cortex (Fig 5A; basal cortex: 0.0314 IDV [0.0262–0.0336]; hippocampus: 0.0037 IDV [0.0023–0.0041], p < 0.001; parietal cortex: 0.0013 IDV [0.0008–0.0023], p<0.001).

Western blot analysis for IL-6 of the same brain regions revealed a significant increase of IL-6 in the basal cortex of SAH animals in comparison to sham animals (SAH 0.023 IDV [0.0165–0.0273]; sham: 0.00011 IDV [0.0001–0.00014] p < 0.001). Comparable to TNF-α, the sham animals also showed higher levels of IL-6 in the hippocampus when compared to SAH animals (SAH: 0.0011 IDV [0.0004–0.0022]; sham: 0.0112 IDV [0.0071–0.0168] p< 0.001). In SAH animals, a significance between the basal cortex and the parietal cortex (basal cortex: 0.023 IDV [0.0165–0.0273]; parietal cortex: 0.0002 IDV [0.0002–0.0003]; p < 0.001) as well as between the basal cortex and the hippocampus (hippocampus: 0.0011 IDV [0.0004–0.00022] p < 0.001) was found. In addition, a significant difference was found between hippocampus and basal cortex (p<0.001) and hippocampus and parietal cortex (parietal cortex: 0.0011 [0.0009–0.0013]; p<0.001) (Fig 5B).

## Discussion

To prove the hypothesis of an HPA-axis activation early after SAH and to evaluate the usability of the main inflammatory markers TNF-α and IL- 6 for brain lesion estimation in the acute phase after SAH, we used an acute SAH model in rats under ongoing anaesthesia, thus avoiding any influence of pain or psychogenic stress on these markers. Our results show rather low values of corticosterone at all sampling points and no difference between sham and SAH animals, pointing against an HPA-axis activation within the first 6h after the insult. The analysis of the inflammatory markers showed significantly stronger increasing levels of IL-6 towards the end of the recording period in the blood of SAH animals. This systemic inflammatory response was confirmed by a significant expression of IL-6 and TNF-α in the basal cortical tissue in close contact with the highest load of extravascular blood in the subarachnoid space.

### Time course of pro-inflammatory cytokines–influence of surgery and early brain injury in SAH

As both important pro-inflammatory cytokines TNF-α and IL-6 have been described to play a role in delayed cerebral vasospasm and brain damage after SAH [7, 8, 10, 22, 23], it is crucial to investigate their expression already within the first hours after the bleeding event according to the concept of early brain injury. In sham animals, IL-6 showed a significant increase only towards the end of the observation period at 6h, suggesting an impact of the surgery itself, which–in the chosen setup—is rather invasive (tracheotomy, cannulation of A. and V. femoralis, skull preparation). Furthermore, comparisons between sham and SAH showed significant differences at 4h and 6h, pointing towards an additional direct and important impact of SAH on IL-6 beyond the surgery itself.

TNF-α showed a comparable time course for both SAH or sham without any significant difference between the groups. Whether the slight fluctuations during the 6h recording phase are significant and probably meaningful early changes of this cytokine in our model has to be proven in further studies with adequately high sample size with a-priori calculation based on the statistic properties of this parameter shown here.

Very little is known about the time course of pro-inflammatory cytokines in the early phase after SAH. Compared to preclinical studies in animals, human data is only available days after the insult [6, 24] with no information on the very first hours after the bleeding. Kato et al investigated the time course of IL-6 and TNF-α in a prechiasmatic blood injection model of SAH in rats, analysed for each time point in separate groups. They showed an IL-6 peak at 1h post SAH and a significant difference to sham up to 6h. TNF-α in their study peaked at one hour in both sham and SAH groups and a significant difference between both groups was found for 3 hours post SAH [9]. Even though the exact time course of IL-6 and TNF-α expression slightly differs from our results, the main pattern of an early increase of inflammatory markers in the blood after SAH appears comparable. The findings of another very recently published study using the endovascular perforation model in rats is in agreement with our findings of no significant changes for TNF-α and a surgery-induced as well as additional SAH specific early elevation of IL-6 [20].Taken together, IL-6 from blood sampling is a very sensitive and robust marker for early SAH induced inflammatory processes, as it was consistently detected in several studies using different models of SAH in rats. The results for TNF-α are more inconsistent, requiring further research with larger sample sizes in each model.

### Markers of inflammation in brain tissue: Mainly in regions with direct blood contact

The expression of IL-6 as well as TNF-α is significantly increased under pathological situations, as shown for ischemic insults in rats and mice [25–27]. In the early phase of SAH, the transient phase of global ischemia induced by the acutely outpouring blood from a ruptured aneurysm (modelled by the injection of blood into the cisterna magna within 1 minute in our model) as well as the molecular cascade started by the extravasated blood components and their degradation products may be responsible for inflammatory responses within the brain tissue. Our data implies that hypoxia, induced by the very early and transiently occurring global ischemia after SAH, may not trigger pro-inflammatory effects in the brain as no increase of IL-6 or TNF-α occurred in the hippocampus, which is most susceptible to ischemic periods compared to other brain regions. Instead, we found a marked increase of IL-6 and TNF-α in tissue samples from the basal cortex of SAH animals, with no change of cytokine expression in the parenchymal tissue of the parietal cortex of SAH compared to sham. As the basal cortex is the area in direct contact with the highest blood load, we hypothesize that inflammation starts primarily due to a local effect of the blood components rather than due to global ischemic or hypoxic events. A recently published study using the endovascular perforation model showed strong TNF-α and IL-6 expression at the RNA level, with IL-6 being significantly expressed only in animals suffering from a strong bleeding which was more pronounced in areas adjacent compared with areas distant to the hematoma at 4 and 6h after the bleeding [20], overall confirming our findings of protein expression of these markers using the blood-injection model.

Interestingly we found higher expression levels of both cytokines in the hippocampus compared to parietal and basal cortical regions in sham animals with a tendency towards even lower expression after SAH. As recently shown by our groups [21, 28], selective neuronal death in hippocampal subareas can be detected very early after SAH, which may at least in part explain the tendency of lower expression levels in SAH animals. However, the unexpected cytokine expression levels in the hippocampus remains unclear and needs further investigation.

### No HPA-axis activation within the first 6h after SAH resembled by corticosterone time course

In our study we did not find a significant difference in the time course of corticosterone between SAH and sham. Of note, even in an animal with a lethal course of SAH we did not find exceeding values different to those from the other SAH and sham animals, strongly arguing against a brain lesion induced HPA-activation in the early hours after SAH. Our study is restricted to the first 6h after SAH. Following focal cerebral ischemia, long-lasting lymphopenia and impaired cytokine expression was only detectable after 12h, suggesting an immunosuppression in the later phase after a focal ischemic insult in the brain [29]. Further studies have to show whether a brain lesion induced HPA-activation also occurs after SAH in the later phase.

Within the acute phase investigated here, all values of corticosterone measured in sham as well as SAH-animals were comparatively low [30]. The first measure of corticosterone showed a slight and probably stress induced corticosterone elevation reflecting a combined restraint, novel environment and anaesthesia-induction stress response. Both sham and SAH animals showed low basal levels thereafter following the surgery and the first hours of the measurement period.

Within the surgery period, the procedure of ear bar fixation is amongst the more painful parts, since we regularly detect a small and transient increase of the systemic blood pressure despite adequate analgesia verified by tail pinch and paw withdrawal reflex. However, we did not see elevated corticosterone levels after ear bar fixation compared with the sampling points before and afterwards. Thus, systemic blood pressure may be an even more sensitive parameter for acute painful events than corticosterone.

In both sham and SAH animals, higher corticosterone values occurred at the end of the observation period at the late afternoon. It is well known that corticosterone follows a circadian rhythm with increasing values measured prior to the active period and rather low concentrations at the end of the active period [31–33]. This circadian rhythm is also nicely monitored in our analysis, with no SAH induced elevation above normal basal levels occurring.

In summary, we have no evidence for an HPA-axis activation, rendering corticosterone a potentially useful stress indicator in the very early phase after SAH. Our data confirms the adequateness of our anaesthesia regime as—apart from the unavoidable but slight elevation due to handling and anaesthesia induction in the morning—the concentrations follow the circadian rhythm and are rather low and noticeable smaller than reported values during relevant stress situations.

### Interaction between corticosterone and markers of inflammation

In our study with an observation period of up to 6h after SAH, we detected an increase of pro-inflammatory cytokines (most conclusive for IL-6) towards the end of the 6h period in SAH animals, pointing to an activation of the pro-inflammatory cascade by the insult with no CNS injury-induced immunodepression taking place at this early phase. This is in line with the results from Prass et al. [29], showing signs of immunodepression only from 12h onwards after focal cerebral ischemia in mice (see discussion above). Further analysis of both pro-inflammatory cytokines and corticosterone in chronic models of SAH have to clarify the validity of corticosterone and inflammatory cytokines for the assessment of the animal’s burden as well as the severity of the brain lesion itself at later time points in animals being allowed to awake.

From animal as well as patients studies it is known that a correlation exists between functional outcome and increased pro-inflammatory cytokines in stroke and SAH [34–36]. For animals, however, up to now it is not known whether the functional impairment induces any burden or suffering as long as the basic needs for survival, housing and social interaction are met, opening an important field for further research to identify sound measures for severity assessment in the chronic phase specifically addressing the neurological deficit following the acute brain lesion.

### Limitations

Power of the study results: Due to a drop-out rate of 31% and one animal not reaching the end of the measuring period, n = 10 animals in SAH and n = 11 animals in sham were finally included in our analysis. These animals were then evenly distributed to the groups for cytokine analysis and corticosterone analysis, ending up with n = 5 SAH and n = 6 sham for cytokines and n = 5 each for corticosterone (Fig 2B). These small numbers may therefore have led to sample size bias with a higher probability of effect inflation [37]. This sample size of 10–11 animals for the ELISA tests was only sufficient to detect an effect size of d = 1.63 with 80% power, which is larger than our measured effect size of 1.1 for IL-6-ELISA at 6h. We are confident that our data are valid, however the statistical analysis of probable differences of markers in blood between sham and SAH and within each group over time have to be interpreted with care. In accordance with our findings, a recently published study [20] used an almost comparable sample size of n = 7 per group and also reported only ambiguous levels of pro-inflammatory cytokines in blood samples. Higher sample size studies have to confirm these results with the chance to even detect smaller differences between the groups, i.e. differences at earlier time points when the cytokines start to rise.

Corticosterone and inflammatory cytokines measured in blood samples from different animals: Due to the limitation of the total amount of blood that can be withdrawn from a rat for repetitive sampling, blood analysis for pro-inflammatory cytokines and corticosterone from the same animal was not possible. Our highly standardized procedure during the surgery and the recording phase leads to only low variation in the relevant parameters throughout the whole experiments, with no differences detected between the cytokine- and corticosterone-groups in the physiological parameters (ABP, blood gases) as well as the parameters characterising the degree and course of the SAH (ICP, CBF, blood load scoring after euthanasia), allowing for comparison of corticosterone with IL-6 and TNF-α at the relevant time points.

### Outlook

In human studies, an increase in pro-inflammatory cytokines can be correlated to outcome in ischemia and haemorrhagic stroke [34, 35]. As our study was specifically designed to evaluate the early changes of inflammatory cytokines and corticosterone in continuously anesthetised animals not suffering from pain or psychogenic stress, we did not perform long-term evaluation of functional outcome or mortality. It would be very interesting and valuable to further investigate whether corticosterone will serve as a valuable marker for animal stress specifically related to SAH compared to the surgery itself in conscious animals at theses early time points. Our here presented results form a valuable basis for these studies in animals being allowed to awake.

### Conclusion

In adequately anaesthetised rats, blood corticosterone showed no significant difference between sham and SAH with low values featuring a circadian rhythm. No evidence for an activation of the HPA-axis was detected, making corticosterone a potentially useful parameter for stress assessment in future chronic studies of SAH. Both groups showed an increase in pro-inflammatory cytokines (most conclusive for IL-6, ambiguous for TNF-α), suggesting an additional, surgery-induced systemic inflammatory response. A local inflammatory reaction was only depicted in the basal cortex, shown by strongly increased protein expression levels of IL-6 and TNF-α in the SAH group. Thus, the pro-inflammatory response seems to start locally in the brain, followed by a systemic response. Biomarkers of inflammation may therefore serve as ideal candidates to assess the severity of the brain lesion independent of further consequences for the animals in respect to suffering, burden and reduced well-being.

## Supporting information

S1 Fig Scoring of blood load of removed and rinsed brains. Score 0: no blood; score 1: only minimal blood; score 2: coverage of basal surface with blood, perivascular blood at least at the origin of larger pial vessels originating from Circle of Willis; score 3: severe blood load on ventral parietal and temporal lobes, perivascular blood following the larger pial vessels along the curvature, blood clots in basal cisterns. Score 0–1: no SAH, sham animals were included as successful when graded with 0 or 1. Score 2–3: successful SAH.
(TIF)

S2 Fig Illustration of brain regions for Western Blot analysis. Tissues encircled: black—hippocampus; blue—basal cortex; green–parietal cortex; only left hemispheres were used.
(TIF)

S3 Fig **Courses of ABP (A, B), CBF (C, D), ICP (E, F).** Early transient hypoperfusion as well as an increase of ICP and ABP is an established effect of SAH, which we were able to confirm in the SAH model used here. Courses of mean values for ABP (A), CBF (C) and ICP (E) for sham (grey) or SAH (black); Box plots of ABP (B), CBF (D) ICP (F) comparing sham (grey) to SAH (black) by repeated-measures 2-way ANOVA followed by Sidak´s multiple comparisons test. * p < 0.05, ** p < 0.01.
(TIF)

S4 Fig Changes of ABP before and after ear- bar insertion. Data were analysed using Mann-Whitney test. * p < 0.05.
(TIF)

S5 Fig Corticosterone concentrations of on animal with a lethal course of SAH. The animal died after time point 120min; despite the lethal course of SAH, the corticosterone concentrations remained rather low and showed an identical course compared with other SAH animals.
(TIF)

S1 Dataset (XLSX)
