# Supplementary figures and images for: Acute changes of pro-inflammatory markers and corticosterone in experimental subarachnoid haemorrhage: A prerequisite for severity assessment
Source: PLoS One. 2019 Jul 30;14(7):e0220467. doi: 10.1371/journal.pone.0220467 (PMC6667150; doi:10.1371/journal.pone.0220467)

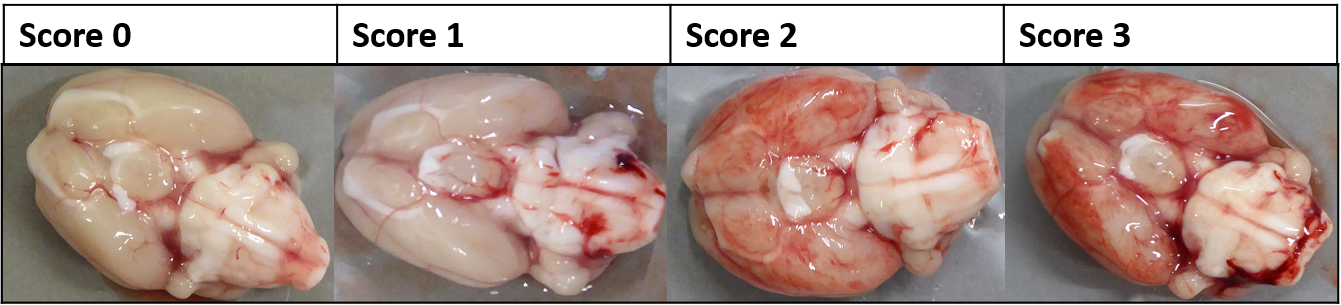

Supplement: S1 Fig — Score 0: no blood; score 1: only minimal blood; score 2: coverage of basal surface with blood, perivascular blood at least at the origin of larger pial vessels originating from Circle of Willis; score 3: severe blood load on ventral parietal and temporal lobes, perivascular blood following the larger pial vessels along the curvature, blood clots in basal cisterns. Score 0–1: no SAH, sham animals were included as successful when graded with 0 or 1. Score 2–3: successful SAH. (TIF) [file pone.0220467.s001.tif]

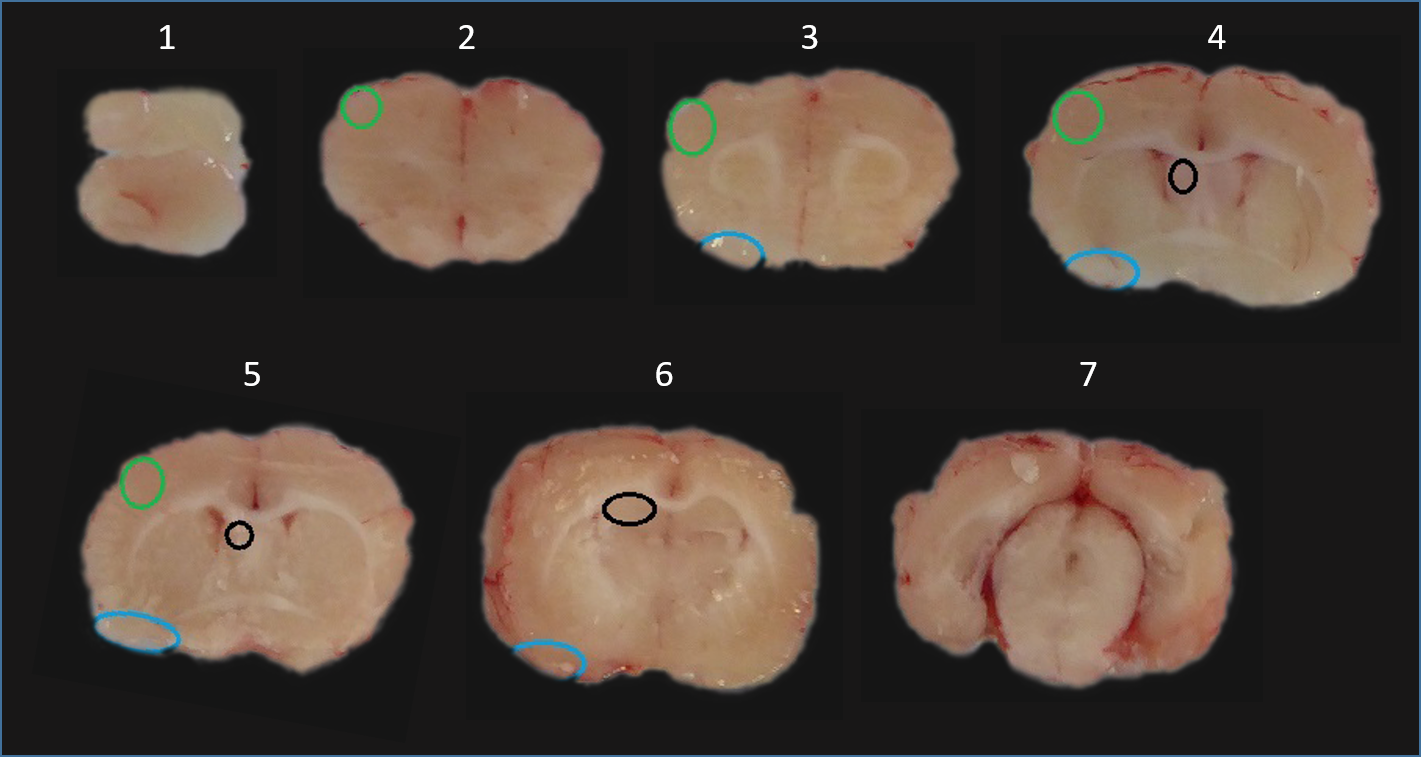

Supplement: S2 Fig — Tissues encircled: black—hippocampus; blue—basal cortex; green–parietal cortex; only left hemispheres were used. (TIF) [file pone.0220467.s002.tif]

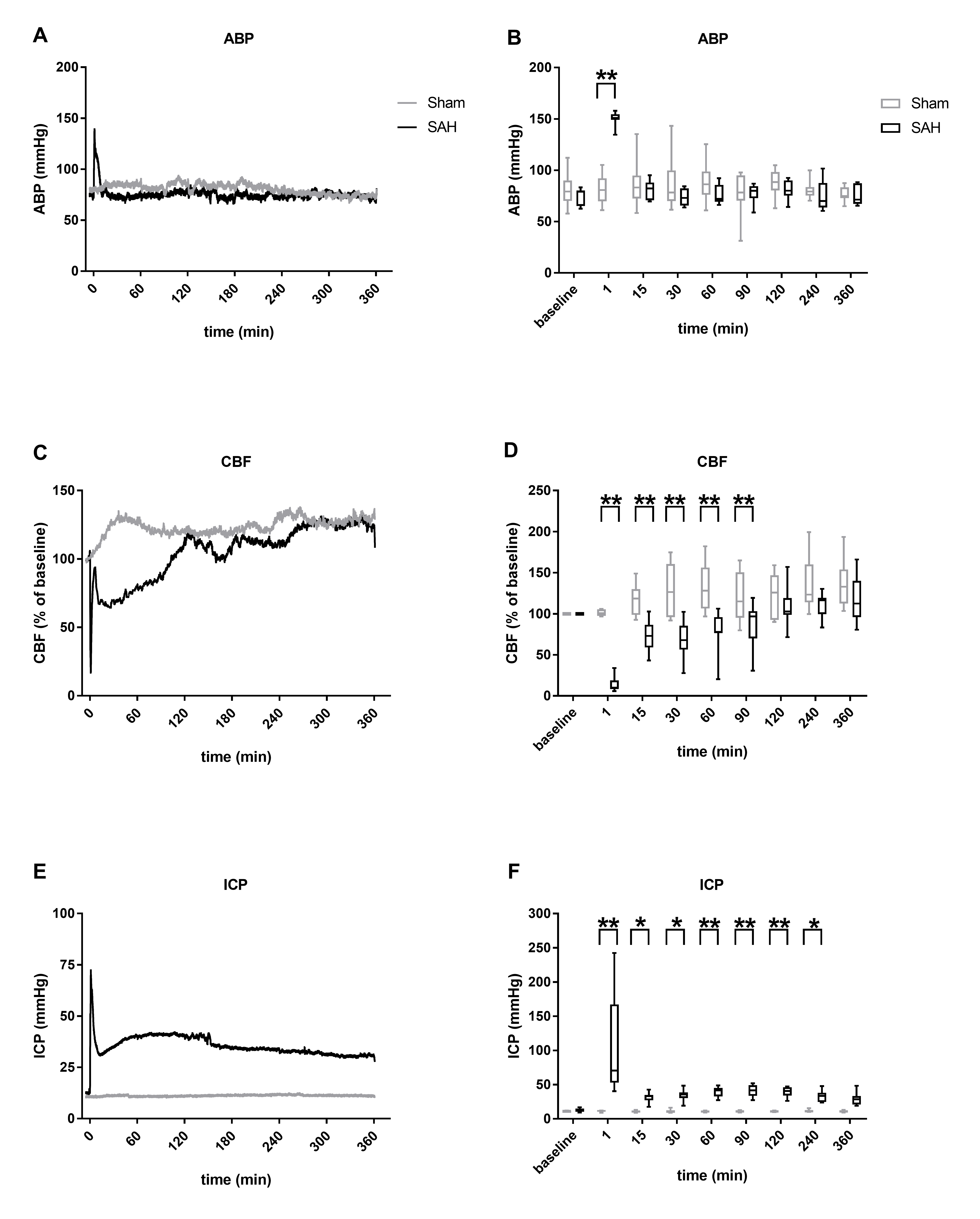

Supplement: S3 Fig — Courses of ABP (A, B), CBF (C, D), ICP (E, F). Early transient hypoperfusion as well as an increase of ICP and ABP is an established effect of SAH, which we were able to confirm in the SAH model used here. Courses of mean values for ABP (A), CBF (C) and ICP (E) for sham (grey) or SAH (black); Box plots of ABP (B), CBF (D) ICP (F) comparing sham (grey) to SAH (black) by repeated-measures 2-way ANOVA followed by Sidak´s multiple comparisons test. * p < 0.05, ** p < 0.01. (TIF) [file pone.0220467.s003.tif]

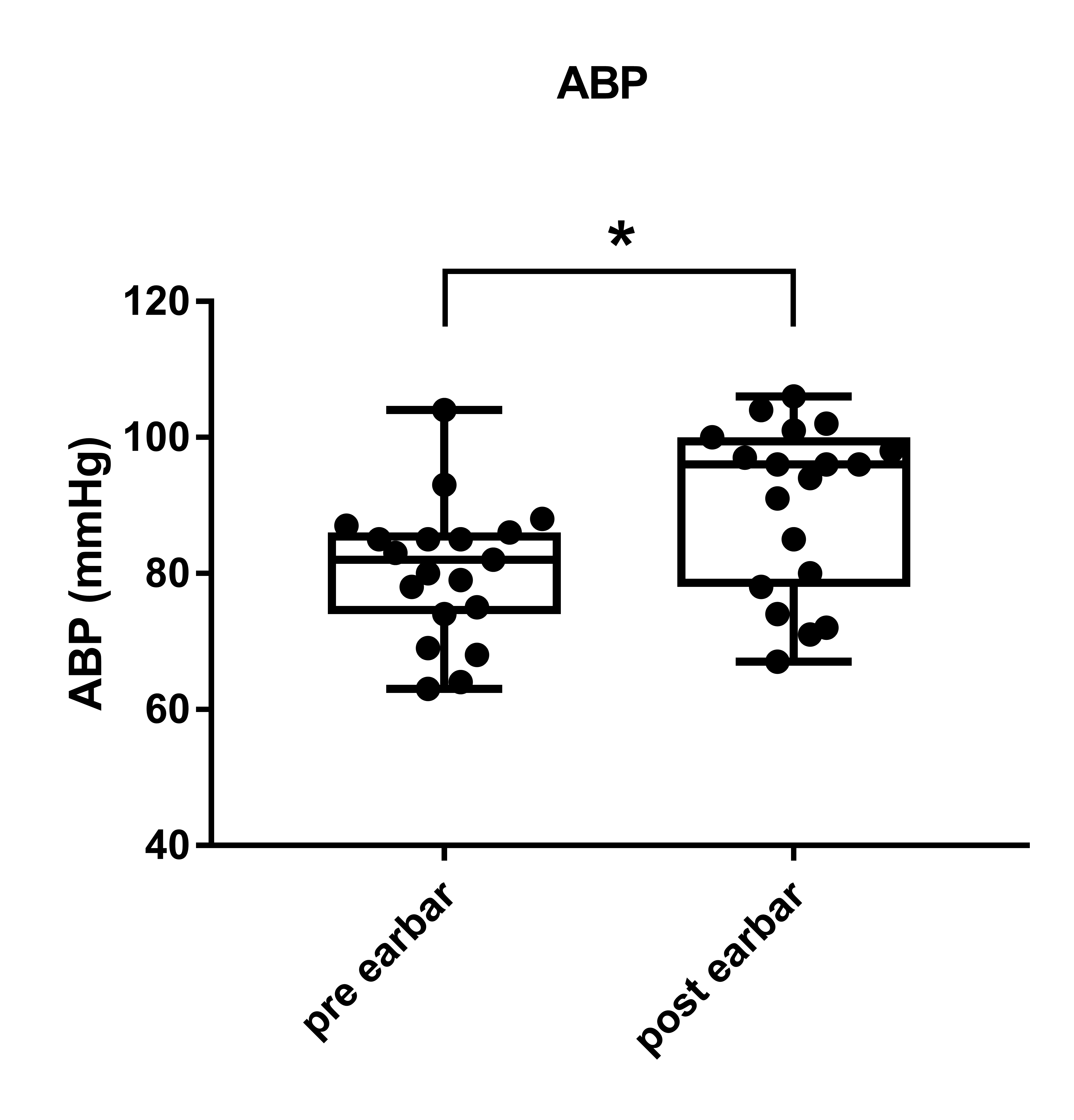

Supplement: S4 Fig — Data were analysed using Mann-Whitney test. * p < 0.05. (TIF) [file pone.0220467.s004.tif]

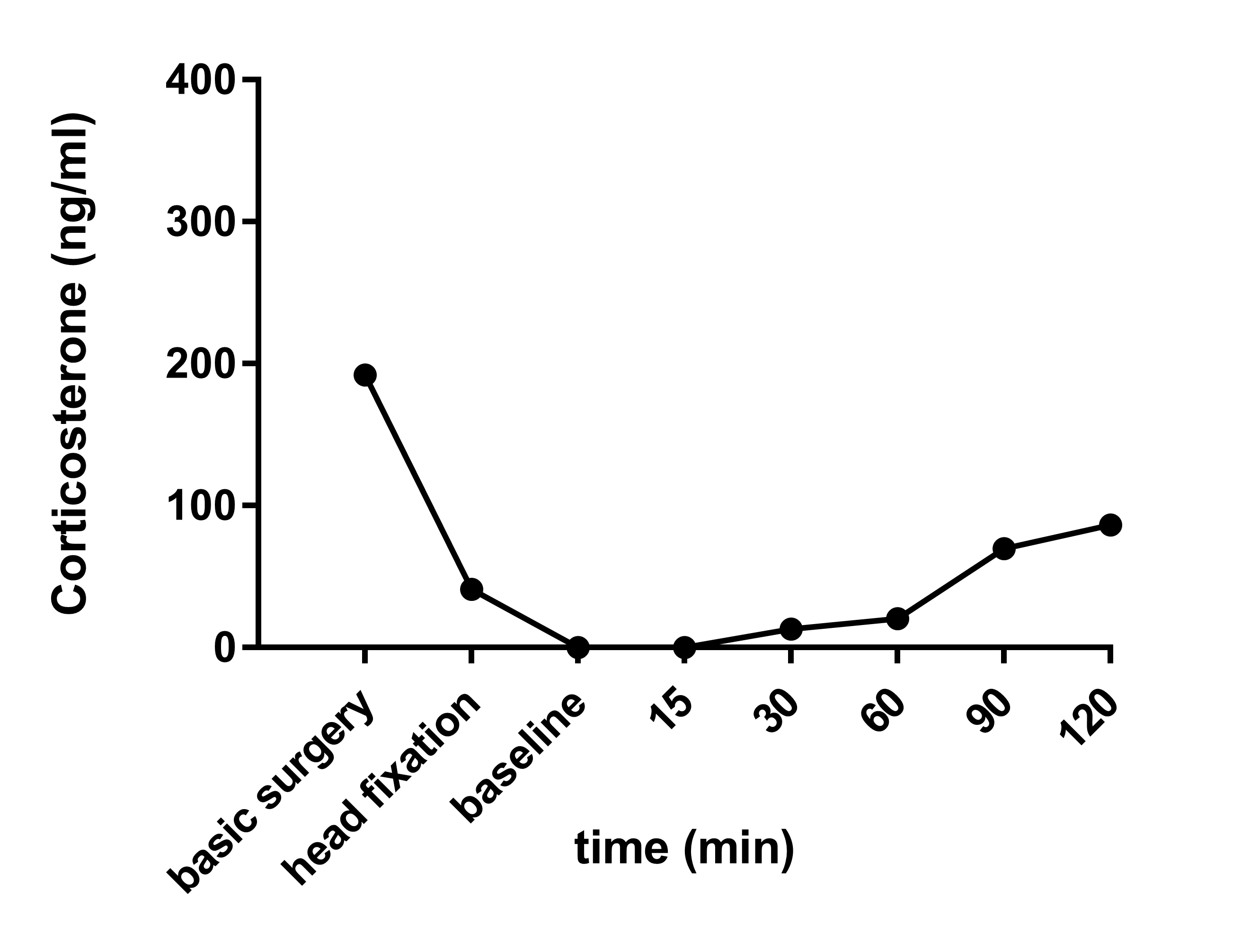

Supplement: S5 Fig — The animal died after time point 120min; despite the lethal course of SAH, the corticosterone concentrations remained rather low and showed an identical course compared with other SAH animals. (TIF) [file pone.0220467.s005.tif]
